# A novel P2X4 receptor-selective antagonist produces anti-allodynic effect in a mouse model of herpetic pain

**DOI:** 10.1038/srep32461

**Published:** 2016-08-31

**Authors:** Yuta Matsumura, Tomohiro Yamashita, Atsushi Sasaki, Eriko Nakata, Keita Kohno, Takahiro Masuda, Hidetoshi Tozaki-Saitoh, Toshiyasu Imai, Yasushi Kuraishi, Makoto Tsuda, Kazuhide Inoue

**Affiliations:** 1 Department of Life Innovation, Graduate School of Pharmaceutical Sciences, Kyushu University, 3-1-1 Maidashi, Higashi-ku, Fukuoka 812-8582, Japan; 2 Department of Molecular and System Pharmacology, Graduate School of Pharmaceutical Sciences, Kyushu University, 3-1-1 Maidashi, Higashi-ku, Fukuoka 812-8582, Japan; 3 Department of Applied Pharmacology, Graduate School of Medicine and Pharmaceutical Sciences, University of Toyama, Toyama 930-0194, Japan; 4 Discovery Research Laboratories, Nippon Chemiphar Co., Ltd., Misato, Saitama 341-0005, Japan; 5 Institute of Neuropathology, University of Freiburg, Neurozentrum, Breisacherstr. 64, Freiburg 79106, Germany

## Abstract

Accumulating evidence indicates that purinergic P2X4 receptors (P2X4R: cation channels activated by extracellular ATP) expressed in spinal microglia are crucial for pathological chronic pain caused by nerve damage, suggesting a potential target for drug discovery. We identified NP-1815-PX (5-[3-(5-thioxo-*4H*-[1,2,4]oxadiazol-3-yl)phenyl]-*1H*-naphtho[1, 2-b][1,4]diazepine-2,4(*3H*,*5H*)-dione) as a novel antagonist selective for P2X4R with high potency and selectivity compared with other P2XR subtypes. In *in vivo* assay for acute and chronic pain, intrathecal administration of NP-1815-PX produced an anti-allodynic effect in mice with traumatic nerve damage without affecting acute nociceptive pain and motor function (although its oral administration did not produce the effect). Furthermore, in a mouse model of herpetic pain, P2X4R upregulation in the spinal cord exclusively occurred in microglia, and intrathecal NP-1815-PX suppressed induction of mechanical allodynia. This model also showed K^+^/Cl^−^ cotransporter 2 (KCC2) downregulation, which is implicated in dorsal horn neuron hyperexcitability; this downregulation was restored by intrathecal treatment with NP-1815-PX or by interfering with brain-derived neurotrophic factor (BDNF) signaling, a P2X4R-activated microglial factor implicated in KCC2 downregulation. Taken together, the newly developed P2X4R antagonist NP-1815-PX produces anti-allodynic effects in chronic pain models without altering acute pain sensitivity, suggesting that microglial P2X4R could be an attractive target for treating chronic pain.

Herpes zoster is caused by the reactivation of varicella zoster virus residing in the sensory ganglion, and is characterized by clustered blisters and severe pain, including mechanical allodynia (pain hypersensitivity to normally innocuous stimuli)^1^. Early treatment of herpes zoster with antiviral agents shortens the duration of skin lesions and complications related to herpes zoster^2^. However, these agents do not promptly relieve herpetic pain^3^. Nonsteroidal anti-inflammatory drugs and antidepressants are used to treat herpes zoster-related pain, but these treatments do not always relieve severe herpetic pain^1^,^4^. Additionally, herpetic pain is a known risk factor for post-herpetic neuralgia^4^,^5^. Thus, the elucidation of the mechanisms underlying induction of herpetic pain and the development of novel analgesics for this pain remain a clinical challenge.

The purinergic P2X receptors, of which seven subtypes (P2X1R-P2X7R) have been identified, are a family of ligand-gated cation channels activated by extracellular ATP^6^,^7^,^8^,^9^. We have shown that expression of the P2X4R subtype is upregulated in microglia, immune cells of the central nervous system, in the spinal dorsal horn (SDH) after peripheral nerve injury, which contributes to the emergence of mechanical allodynia^10^,^11^,^12^,^13^,^14^. Furthermore, in a wide range of assays to measure physiological and pathological pain of P2X4R-deficient mice, a predominant mouse phenotype exhibits reduced mechanical allodynia caused by nerve damage^15^,^16^, supporting that microglial P2X4R is involved in nerve damage-induced chronic pain. Recent studies using a herpetic pain model in which herpes simplex virus type 1 (HSV-1) was inoculated on the mouse skin^17^,^18^ revealed nerve damage in dorsal root ganglion (DRG) neurons^19^ and activated microglia in the spinal cord^20^. Activation of the spinal microglia correlates with the induction time course of herpetic allodynia^20^, which raises the possibility that activated spinal microglia in HSV-1-inoculated mice may induce P2X4R expression and thereby contribute to herpetic allodynia.

Recently, some compounds have been identified as P2X4R antagonists^21^, such as the benzodiazepine derivative 5-(3-bromophenyl)-1,3-dihydro-2*H*-benzofuro[3,2-*e*]-1, 4-diazepin-2-one (5-BDBD)^22^,^23^. However, the potency of this compound to inhibit P2X4R was similar to TNP-ATP, a non-selective P2X antagonist, and its selectivity for P2X4R versus other P2XR subtypes remained unknown^21^. The compound also displayed very low water solubility^21^. Two N-substituted phenoxazines–PSB-12054 [N-(benzyloxycarbonyl)phenoxazine] and PSB-12062 [N-(p-methylphenyl)sulfonylphenoxazine]–were developed as noncompetitive antagonists of P2X4R^24^. The former potently and selectively inhibited human P2X4R compared with human P2X2R, P2X3R, and P2X7R, but was less potent on rat and mouse P2X4Rs. The latter exhibited moderate potency (IC_50_: approximately 1 μM) for these species and a selectivity similar to PSB-12054. These compounds also possessed low water solubility^21^. The phenylurea BX430 (1-(2,6-dibromo-4-isopropyl-phenyl)-3-(3-pyridyl)urea) exhibited antagonistic properties on human P2X4R with submicromolar potency in a noncompetitive allosteric manner^25^. However, BX430 exhibited no effect on rat and mouse P2X4Rs. As such, P2X4R-selective antagonists with high potency, no species-restricted effect, water-solubility and, most importantly, an anti-allodynic effect in rodent chronic pain models remains to be identified.

Results from screening a chemical library in the present study identified the compound NP-1815-PX (5-[3-(5-thioxo-*4H*-[1,2,4]oxadiazol-3-yl)phenyl]-*1H*-naphtho[1,2-b][1,4]diazepine-2,4(*3H*,*5H*)-dione) as a novel P2X4R antagonist. NP-1815-PX was selective for human P2X4R versus other P2XRs tested, with high potency and water solubility. The inhibitory effect of NP-1815-PX was also shown on rat and mouse P2X4Rs. Using the newly developed P2X4R antagonist, we showed that intrathecal administration of NP-1815-PX alleviated nerve damage-associated mechanical allodynia in chronic pain models of spinal nerve transection and by inoculation of HSV-1 (herpetic pain model) without altering acute pain sensitivity. Taken together, the novel P2X4R antagonist NP-1815-PX produces anti-allodynic effects in chronic pain models, which supports our hypothesis that microglial P2X4R could be a potential target for treating chronic pain.

## Results

### Selective inhibition of P2X4R by NP-1815-PX

**Figure 1:**
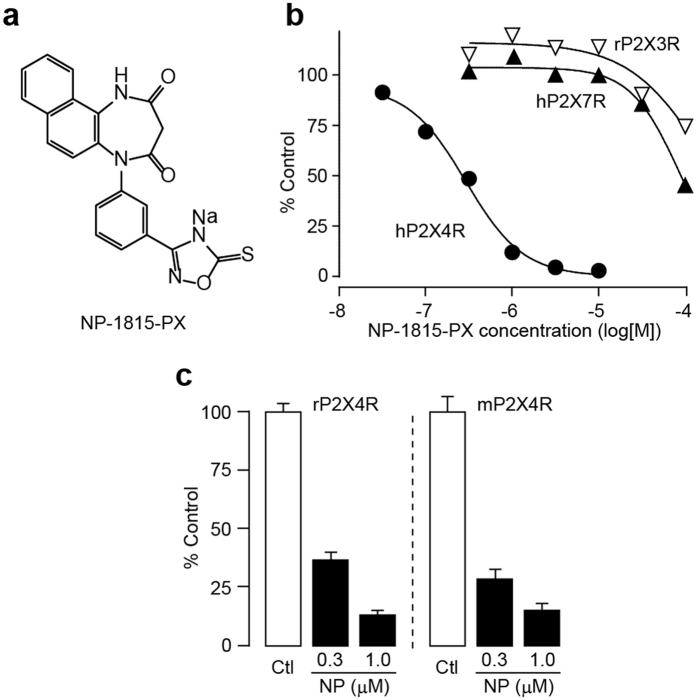
NP-1815-PX is a novel P2X4R-selective antagonist. (**a**) Chemical structure of NP-1815-PX. (**b**) Effect of NP-1815-PX on agonist-induced intracellular Ca^2+^ ([Ca^2+^]_i_) responses in 1321N1 cells expressing either hP2X4R, rP2X3R, or hP2X7R. Each plot indicates the effect of NP-1815-PX at various concentrations (30 nM–100 μM) as the percentage of control (P2X agonist alone-induced [Ca^2+^]_i_ responses: 1 μM ATP for hP2X4R, 0.3 μM ATP for rP2X3R, and 10 μM BzATP for hP2X7R). Pretreatment with NP-1815-PX 15 min prior to application of ATP or BzATP. (**c**) Inhibitory effect of NP-1815-PX on rat and mouse P2X4R (rP2X4R and mP2X4R, respectively)-mediated [Ca^2+^]_i_ responses evoked by ATP (10 μM) in 1321N1 cells. Pretreatment with NP-1815-PX (NP: 0.3 and 1 μM) 10 min prior to application of ATP. Data represent mean ± SEM.

To identify small molecules that inhibit P2X4R, we initially screened a chemical library using real-time Ca^2+^ imaging in 1321N1 human astrocytoma cells stably expressing human P2X4Rs (hP2X4R-1321N1). Native 1321N1 cells did not show any changes in intracellular Ca^2+^ levels ([Ca^2+^]_i_) evoked by ATP application (data not shown). We identified the compound NP-1815-PX (Fig. 1a), which potently inhibited the P2X4R-mediated [Ca^2+^]_i_ increase in hP2X4R-1321N1 cells (Fig. 1b). The inhibitory effect of NP-1815-PX was concentration-dependent, with an IC_50_ value of 0.26 μM for hP2X4R. Conversely, 1 μM of NP-1815-PX had no effect on ATP-induced [Ca^2+^]_i_ increase in hP2X7R- and rP2X3R-1321N1 cells (Fig. 1b). The IC_50_ values for other P2XR subtypes were >30 μM for hP2X1R, rat (r) P2X3R, hP2X2/3R and hP2X7R, and 7.3 μM for hP2X2R, indicating that NP-1815-PX is highly selective for P2X4R compared with other P2XRs. Additionally, a similar potent inhibitory effect of NP-1815-PX was detected in 1321N1 cells stably expressing rP2X4R and mouse (m) P2X4R (Fig. 1c). Furthermore, NP-1815-PX was easily dissolved in water (>100 mg/mL).

**Figure 2:**
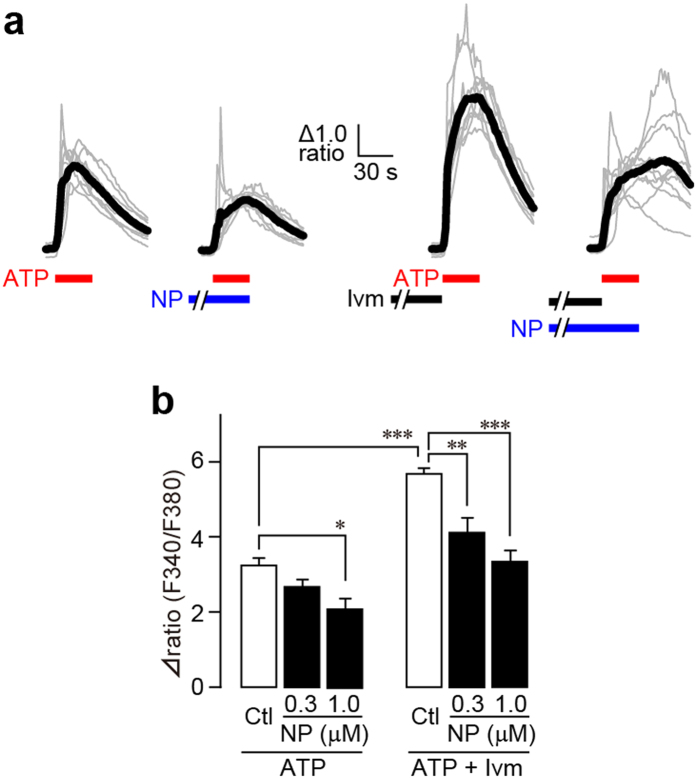
NP-1815-PX inhibits ATP-mediated responses in primary cultured microglial cells. (**a**) Representative traces of [Ca^2+^]_i_ increases in response to ATP (50 μM for 30 sec, red horizontal bars) in primary cultured microglial cells. Treatment with NP-1815-PX (NP: 1 μM for 10 min) and with ivermectin (Ivm: 3 μM for 3 min) are shown as blue and closed horizontal bars, respectively. (**b**) Each column shows the relative increase ratio (F340/F380) from the basal level prior to ATP application with or without NP-1815-PX (NP: 0.3 and 1 μM) in primary cultured microglial cells (n = 5–8). Data represent mean ± SEM. *P < 0.05, **P < 0.01, ***P < 0.001.

We then tested whether NP-1815-PX exhibits an inhibitory effect on microglial P2X4R, whose activity is crucial for mechanical allodynia after nerve injury^10^,^11^,^12^,^13^,^14^. NP-1815-PX (1 μM) significantly reduced the ATP (50 μM)-induced [Ca^2+^]_i_ increase in primary cultured microglial cells (Fig. 2a,b). Previous studies have shown that rat primary cultured microglia express not only P2X4R, but also other P2 receptor subtypes that produce a [Ca^2+^]_i_ increase in response to ATP or other nucleotides^10^,^26^,^27^. To selectively identify a P2X4R-mediated component in the ATP-induced [Ca^2+^]_i_ responses, we used ivermectin, an antibiotic known to be a positive allosteric modulator specific for P2X4R^28^. Pre-incubation of microglial cells with ivermectin clearly potentiated the ATP-induced [Ca^2+^]_i_ responses (Fig. 2a,b). NP-1815-PX also suppressed this enhancement in a dose-dependent manner; 1 μM NP-1815-PX almost completely abolished the ivermectin-induced potentiation. Besides P2X4R, P2X7R is also expressed in microglia. However, [Ca^2+^]_i_ responses in response to 3′-O-(4-benzoyl)benzoyl-ATP (BzATP), an agonist for P2X7R, was not suppressed by 1 μM NP-1815-PX in BV-2, a cell line of mouse microglia (F340/F380 value: control, 1.61 ± 0.06, n = 70 cells; NP-1815-PX: 1.64 ± 0.09, n = 69 cells).

### NP-1815-PX effects on mechanical allodynia after traumatic nerve damage

**Figure 3:**
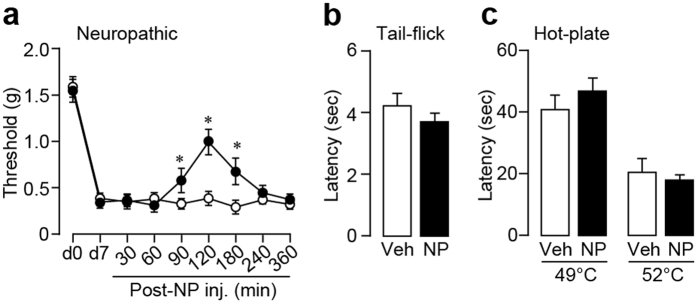
NP-1815-PX reduces mechanical allodynia by traumatic nerve injury. (**a**) The withdrawal threshold (grams) of mechanical stimulation by von Frey filaments applied to the mouse hindpaw before (d0) and 7 days (d7) after spinal nerve transection. NP-1815-PX (30 pmol/5 μl) or vehicle (Veh: PBS, 5 μl) was intrathecally administered to nerve-transected mice on day 7 and the paw withdrawal threshold was measured for 360 min after the injection. *P < 0.01 vs. vehicle-treated mice. n = 5–8. (**b**,**c**) Tail-flick (**b**) and hot-plate (**c**) tests. NP-1815-PX or vehicle (Veh: PBS, 5 μl) was intrathecally administered to normal mice 120 min before testing. Values represent latencies to flick their tail from the heat source (n = 6–7, **b**), and latencies for animals to lick their hindpaws or jump (n = 6–7, **c**). Data represent mean ± SEM.

We examined the effects of NP-1815-PX on mechanical allodynia in a chronic pain model in the mouse, which was generated by transecting the 4th lumbar spinal nerve. Consistent with previous studies^13^,^14^,^29^, spinal nerve transection produced a profound decrease in the withdrawal threshold of the hindpaw at 7 days post-surgery (Fig. 3a). Intrathecal administration of NP-1815-PX on day 7 resulted in a significant amelioration of the decreased threshold. This suppressive effect could be occasionally misinterpreted as a result of non-specific motor dysfunction. However, in the rotarod performance test, NP-1815-PX produced no change in time on the rotarod (PBS: 60.0 ± 0.0 sec, n = 6; NP-1815-PX: 60.0 ± 0.0 sec, n = 7). We also tested acute physiological pain responses in tail-flick and hot-plate tests, revealing that intrathecal NP-1815-PX had no effect on latencies for the mice to flick their tails (Fig. 3b) and hindpaws (Fig. 3c) away from heat stimulus. These results indicates that NP-1815-PX has an inhibitory effect on nerve damage-induced mechanical allodynia without any alterations in basal pain sensitivity or motor function.

### Anti-allodynic effect of NP-1815-PX in a herpetic pain model

**Figure 4:**
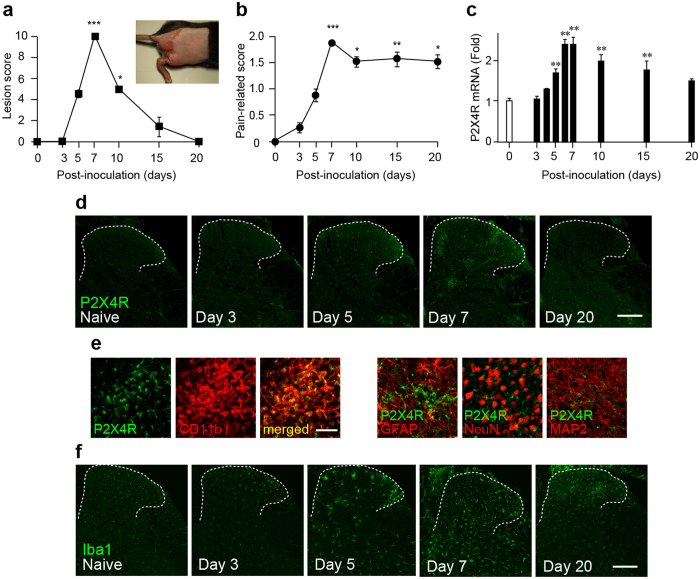
Increased P2X4R expression in spinal microglia in a herpetic pain model. (**a,b**) Time-course of skin lesion (**a**) and mechanical allodynia (**b**) after HSV-1 inoculation. Skin lesions (inset picture) were assessed with a lesion score before and after HSV-1 inoculation on the skin of mice (**a**). Mechanical allodynia was assessed as a pain-related score following light brushing of the plantar surface of the hindpaw (**b**). Data represent mean ± SEM [n = 6, *P < 0.05, **P < 0.01, ***P < 0.001 vs. normal mice (day 0)]. (**c**) Time-course of P2X4R mRNA expression in total RNA extracted from the spinal dorsal horn before and after HSV-1 inoculation. Values represent the relative ratio of P2X4R mRNA (normalized to GAPDH mRNA value) to normal mice. Data represent mean ± SEM [n = 4–5, **P < 0.01 vs. normal mice (day 0)]. (**d**) Representative images of P2X4R immunofluorescence in the spinal dorsal horn before and after HSV-1 inoculation (scale bar, 200 μm). (**e**) Double immunolabeling of P2X4R with CD11b (a marker of microglia), GFAP (a marker of astrocytes), NeuN, or MAP-2 (markers of neurons) in the spinal dorsal horn 7 days after HSV-1 inoculation (scale bar, 50 μm). (**f**) Iba1 immunofluorescence in the spinal dorsal horn before and after HSV-1 inoculation (scale bar, 200 μm).

To determine the anti-allodynic effect of NP-1815-PX under another chronic pain condition, we used a model of herpetic pain. HSV-1 was inoculated on the skin of mice, which has been shown to induce nerve damage in DRG neurons^19^ and produce mechanical allodynia^17^,^18^,^30^. Herpes zoster-like skin lesions developed at day 5, peaked at day 7, and were completely healed by day 20 (Fig. 4a). Also, the pain-related scores in response to brush stroking gradually increased from 3 to 5 days and reached a peak at 7 days (Fig. 4b), implying the induction of mechanical allodynia. We then examined P2X4R mRNA expression in the SDH by quantitative PCR analysis. Expression of P2X4R mRNA progressively increased in the HSV-1 inoculated mice, with greatest expression on day 7 (Fig. 4c). Over the next 13 days (day 20), the increased levels of P2X4R mRNA gradually decreased (day 20). At the protein level, we observed increased P2X4R immunofluorescence in the SDH at 7 days after HSV-1 inoculation (Fig. 4d). P2X4R-positive cells expressed CD11b, a marker for macrophage/microglia (Fig. 4e), but not the neuronal markers NeuN and MAP2, nor the astrocytic marker GFAP (Fig. 4e). Immunohistochemical analysis also showed a change in spinal microglia morphology from ramified to a round shape (Fig. 4f), which was consistent with previous results^20^.

**Figure 5:**
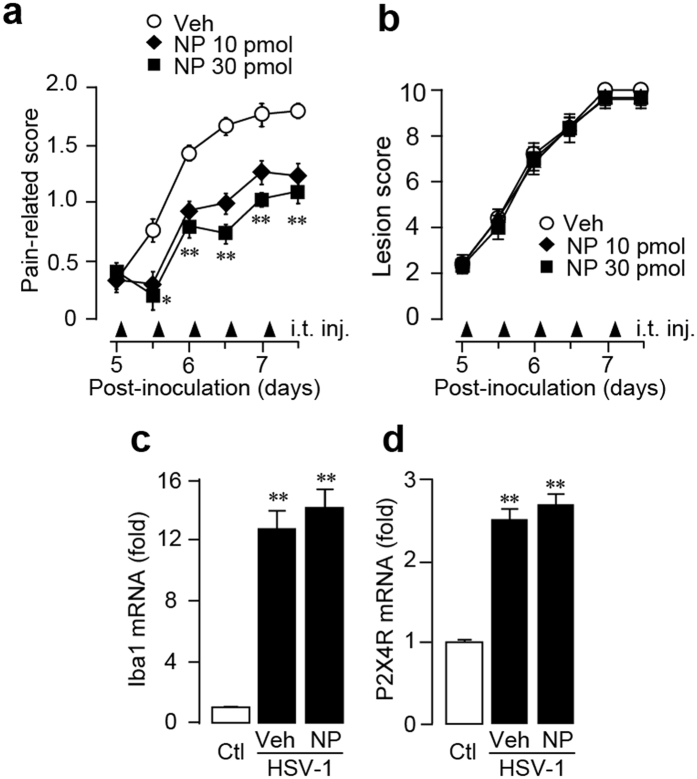
Anti-allodynic effect of NP-1815-PX in herpetic pain model. (**a,b**) Effect of intrathecally administered NP-1815-PX on induction of HSV-1-evoked mechanical allodynia (**a**) and skin lesions (**b**) on day 5 to 7 after HSV-1 inoculation in mice. Mechanical allodynia was assessed with a pain-related score following light brushing of the hindpaw. Skin lesions were assessed with a lesion score. The pain-related scores and skin lesion scores were examined at day 5 to 7. NP-1815-PX or vehicle (Veh: PBS) was intrathecally administered (i.t. inj.) twice daily from day 5 to 7 (closed triangles indicated on horizontal X-axis). Data represent mean ± SEM (n = 5–6, *P < 0.05, **P < 0.01 vs. vehicle control group). (**c,d**) Real-time PCR analysis of Iba1 and P2X4R mRNA expression in total RNA extracted from the spinal dorsal horn (day 6) in normal mice (Ctl) and in HSV-1-inoculated mice intrathecally treated with vehicle (Veh: PBS) or NP-1815-PX. Values represent the relative ratio of Iba1 and P2X4R mRNA expression (normalized to GAPDH mRNA value) to normal mice (Ctl). Data represent mean ± SEM [n = 3–4, **P < 0.01 vs. normal mice (Ctl)].

To test the effect of NP-1815-PX on herpetic allodynia, we intrathecally administrated NP-1815-PX twice daily from day 5 to 7 after HSV-1 inoculation. NP-1815-PX (10 and 30 pmol/mouse) dose-dependently decreased the pain-related score, and induction of mechanical allodynia was suppressed (Fig. 5a). However, the skin lesions remained unchanged compared with vehicle-treated HSV-1 mice (Fig. 5b). Additionally, neither Iba1 nor P2X4R expression was altered by intrathecal NP-1815-PX (Fig. 5c,d). These results suggested that NP-1815-PX exhibits an inhibitory effect on mechanical allodynia in a model of herpetic pain.

**Figure 6:**
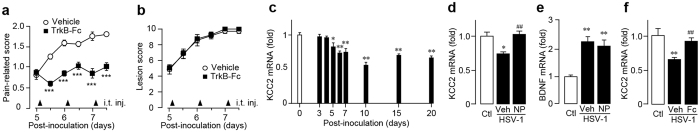
Role of the P2X4R-BDNF-KCC2 pathway in herpetic allodynia. (**a,b**) Effect of intrathecally administered TrkB-Fc chimeric protein on induction of HSV-1-evoked mechanical allodynia in mice. Mechanical allodynia was assessed with a pain-related score following light brushing of the hindpaw (**a**). Skin lesions were assessed with a lesion score (**b**). The pain-related scores and skin lesion scores were examined from day 5 to 7 after viral inoculation. Following behavioral measurements on each day, TrkB-Fc (100 ng/mouse) or vehicle was intrathecally administered (i.t. inj.) once daily from day 5 to 7 to HSV-1-inoculated mice (closed triangles indicated on horizontal X-axis). Data represent mean ± SEM (n = 7–8, *P < 0.05, **P < 0.01, vs. vehicle control group). (**c**) Time-course of KCC2 mRNA expression in total RNA extracted from the spinal dorsal horn before and after HSV-1 inoculation by real-time PCR analysis. Values represent the relative ratio of KCC2 mRNA (normalized to GAPDH mRNA value) to normal mice (Ctl) value. Data represent mean ± SEM [n = 4–5, *P < 0.05, **P < 0.01 vs. normal mice (day 0)]. (**d,e**) Changes in expression of KCC2 (**d**) and BDNF (**e**) in the spinal dorsal horn (day 6) of normal mice (Ctl) and of HSV-1-inoculated mice intrathecally treated with vehicle (Veh: PBS) or NP-1815-PX (NP: three times from day 5 to 6), respectively. Values represent the relative ratio of BDNF and KCC2 mRNA expression (normalized to GAPDH mRNA value) to normal mice. Data represent mean ± SEM (n = 3–4, *P < 0.05, **P < 0.01 vs. normal control mice; ^##^P < 0.01 vs. vehicle-treated HSV-1 mice). (**f**) HSV-1-induced KCC2 downregulation in the spinal dorsal horn (day 6) of normal mice (Ctl) and HSV-1-inoculated mice intrathecally treated with vehicle (Veh) or TrkB-Fc (Fc: once a day at day 5), respectively. Values represent the relative ratio of KCC2 mRNA expression (normalized to GAPDH mRNA value) to naive mice (Ctl). Data represent mean ± SEM [n = 3–6, **P < 0.01 vs. normal mice (Ctl); ^##^P < 0.01 vs. vehicle-treated HSV-1 mice].

Previous studies demonstrated that when BDNF is released from ATP-stimulated microglia via the P2X4R signaling pathway^31^, it activates the TrkB receptor and subsequently downregulates KCC2 expression in SDH neurons^12^, which has been implicated in neuronal hyperexcitability resulting from altered Cl^−^ extrusion and in allodynia^32^,^33^. To determine the role of the P2X4R signaling pathway associated with BDNF and KCC2 in herpetic pain, we intrathecally administrated recombinant TrkB-Fc chimera protein, which sequesters BDNF^12^,^34^, once daily from day 5 to 7 post-inoculation. TrkB-Fc (100 ng/mouse), a dose known to suppress allodynia in other chronic pain models^35^, effectively suppressed the HSV-1-induced mechanical allodynia (Fig. 6a) without any effect on the skin lesions (Fig. 6b). We also found that KCC2 mRNA expression in the SDH significantly decreased at around 1 week post-inoculation and lasted until at least day 20 (Fig. 6c). Interestingly, intrathecal NP-1815-PX from day 5 to 6 prevented downregulation of KCC2 mRNA expression in the SDH (Fig. 6d) without altering the increased BDNF expression (Fig. 6e). Moreover, intrathecal treatment with TrkB-Fc abolished the HSV-induced KCC2 downregulation (Fig. 6f). Taken together, these data suggested that the spinal P2X4-BDNF-KCC2 pathway may be involved in induction of herpetic pain.

## Discussion

Some P2X4R antagonists have been developed in previous studies^21^, but there were drawbacks in potency, selectivity, and water solubility (see Introduction)^21^. In the present study, we identified NP-1815-PX as a novel P2X4R antagonist that resolved these problems. NP-1815-PX was easily dissolved in water and inhibited rodent and human P2X4Rs with a high potency. Among the P2XRs tested, this compound was selective for P2X4R. Most importantly, NP-1815-PX was the first P2X4R antagonist to produce an anti-allodynic effect in pathological chronic pain models. Additionally, when NP-1815-PX was intrathecally administered, it did not affect the behavioral responses in assays for acute physiological pain or motor coordination, which suggests a predicted therapeutic benefit of this antagonist. Thus, we provide evidence showing that the selective P2X4R antagonist NP-1815-PX alleviates pain hypersensitivity caused by nerve damage without affecting normal pain sensitivity or motor function.

This study also revealed that in the herpetic pain model, P2X4R expression was upregulated in the SDH, with expression exclusive to microglia. The morphology of spinal microglia in HSV-1-inoculated mice became altered from a ramified to a hypertrophic or amoeboid shape, and their numbers increased^20^, both of which are morphological features of activated microglia. The time-course of P2X4R upregulation in spinal microglia paralleled the induction of HSV-1-induced mechanical allodynia, and intrathecal administration of NP-1815-PX suppressed the allodynia induction, suggesting that P2X4R activation in spinal microglia is involved in the induction of herpetic pain. To determine the necessity of microglial P2X4R in herpetic pain is an important issue in the future that should be investigated by a genetic approach (e.g., *Cx3cr1*^CreERT^; *P2rx4*^flox/flox^ mice) for manipulating P2X4R gene in a microglia-selective manner^36^,^37^.

We have previously reported that P2X4R activation in spinal microglia leads to the release of bioactive factors, such as BDNF, which has been implicated in the hyperexcitability of SDH neurons by reducing inhibition and converting GABA_A_ receptor-mediated inhibition to excitation^10^,^12^,^32^. This microglia-to-neuron P2X4R-BDNF-KCC2 pathway in the SDH may also be involved in herpetic pain. Indeed, we demonstrated that KCC2 expression in the SDH decreased after HSV-1 inoculation, and this was prevented by intrathecal treatment with either the P2X4R antagonist NP-1815-PX or TrkB-Fc chimera protein. Similar to the anti-allodynic effect of NP-1815-PX, the pharmacological blockade of spinal BDNF signaling by TrkB-Fc chimera protein suppressed induction of herpetic allodynia. These findings support the role for P2X4R in microglial BDNF release^32^. P2X4R has also been reported to be involved in BDNF synthesis in cultured microglial cells^31^. However, the transient upregulation of BDNF expression in the SDH of HSV-1 mice (although the type of cells upregulating BDNF remains unclear) was not suppressed by intrathecal NP-1815-PX. This suggests that spinal BDNF upregulation by HSV-1 inoculation may be independent of the P2X4R-mediated signaling pathway. Nevertheless, we cannot exclude the possibility that the NP-1815-PX doses used in the present study may not be sufficient to block spinal BDNF upregulation *in vivo,* because NP-1815-PX exhibits potency in the submicromolar concentration range. Thus, the development of more potent P2X4R-selective antagonists would help to address this issue. Additionally, one drawback of NP-1815-PX is its low ability to cross the blood-brain barrier (data not shown). Oral administration of this compound had no effect on mechanical allodynia after spinal nerve injury in rats (data not shown; we thus did not investigate pharmacokinetic properties of NP-1815-PX). Therefore, it is important to address penetration across the blood-brain-barrier in future studies identifying novel P2X4R-selective antagonists.

It should also be noted that the induction of herpetic allodynia was not completely suppressed by intrathecal NP-1815-PX, suggesting that another pathway might contribute to induction of herpetic pain. This includes a pathway via other purinergic receptor subtypes expressed in microglia, because we found that P2X7R, P2Y6R, and P2Y12R were also upregulated in the SDH of HSV-1-inoculated mice (data not shown). P2X7R and P2Y12R have been implicated in nerve injury-induced mechanical allodynia^38^,^39^, and pharmacological blockades of these receptors produce anti-allodynic effects^39^,^40^,^41^,^42^,^43^,^44^. Alternatively, a recent study has shown that traumatic nerve injury-induced upregulation of spinal cord P2X4R is not induced in female mice and that mechanical allodynia in female mice involves adaptive immune cells, such as T cells^45^, which is independent on microglial P2X4R. In the present study, only female mice were used, and many CD3-positive T cells have been reported to be observed in the spinal cord of the model of herpetic pain^20^. Therefore, such a P2X4R-independent signaling might also be involved in herpetic allodynia. However, a more recent work has shown that microglia equally participate in neuropathic pain development in both male and female mice^46^, and thus a sex difference in the contribution of microglia in neuropathic pain seems to remain controversial.

The anti-allodynic effect of TrkB-Fc chimera protein was much more effective than that NP-1815-PX and the reasons for this remain unclear. However, we have considered the possible involvement of other cell types expressing BDNF. Indeed, primary afferent sensory neurons with central terminals located in the SDH also upregulate BDNF expression following peripheral tissue inflammation^47^, and BDNF has been shown to play an important role in regulating inflammatory pain, but not neuropathic pain^47^. Because skin inflammation was evident in the HSV-1-inoculated mice, it is conceivable that spinal BDNF derived from primary afferent sensory neurons, which is presumably independent of microglial P2X4R, might play a role in HSV-1-induced herpetic pain.

In summary, our study identified NP-1815-PX as a novel P2X4R-selective antagonist with high potency, selectivity compared with other P2XRs, and water solubility. NP-1815-PX produced anti-allodynic effects in chronic pain models. In particular, our findings not only provide evidence for a new mechanism underlying induction of herpetic allodynia, but also suggest a novel therapeutic approach to this pain. Herpetic pain is a known risk factor for post-herpetic neuralgia, a condition where severe pain persists for an extended period of time after herpes zoster infection^4^,^5^. Future studies are needed to determine whether the pharmacological blockade of microglial P2X4Rs in the SDH during an induction phase of herpetic pain can also effectively prevent the progression to post-herpetic neuralgia. It will also be beneficial to identify a P2X4R-selective antagonist that is effective via oral administration.

## Methods

### Screening for P2X4R antagonist

1321N1 human astrocytoma cells stably expressing each P2X receptor subtype (rat (r), mouse (m), and human (h) P2X4R as well as other subtype) were used. P2XR-expressing 1321N1 cells were maintained in Dulbecco’s-modified Eagle’s medium (DMEM) supplemented with 10% fetal bovine serum in a humidified atmosphere of 5% CO_2_ at 37 °C. To identify hP2X4R antagonists, a cell-based assay was performed in 96-well plates to measure intracellular Ca^2+^concentrations ([Ca^2+^]_i_). The Ca^2+^ indicator Fura2-AM, which was dissolved in a balanced salt solution (BSS; composition in mM: NaCl 150, KCl 5, CaCl_2_ 1.8, MgCl_2_ 1.2, D-glucose 10, and HEPES 25; pH 7.4), was loaded onto the cells for 45 min at room temperature. Then, the cells were washed with BSS and incubated in the presence or absence of compounds for 15 min. Fluorescence in the individual wells was measured using FLUOstar Optima (BMG Labtech) or EnVisionXcite (ParkinElmer) plate readers. Fluorescence changes after application of ATP (1 and 0.3 μM for hP2X4R and rP2X3R, respectively) or BzATP (10 μM for hP2X7R) were monitored, and the fluorescence ratio was calculated (F340/F380) as the index of intracellular Ca^2+^ responses. IC_50_ values were determined by nonlinear regression in GraphPad Prism 5 (GraphPad Software, San Diego, CA). For testing in rP2X3R-expressing cells, 3 μM cibacron blue, a positive allosteric modulator of P2X3R, was co-applied with NP-1815-PX to increase the recovery rate from agonist-induced desensitization of P2X3R^48^. In the hP2X7R antagonism test, a modified assay buffer was used (in mM: Na-glutamate 150, KCl 5, CaCl_2_ 0.5, MgCl_2_ 0.1, D-glucose 10, and HEPES 25; pH 7.4). The effects of NP-1815-PX on hP2X1R, hP2X2R, and hP2X2/hP2X3R were similarly assessed by Ca^2+^ imaging, which was outsourced to ChanTest Corporation (Cleveland, OH). To evaluate rP2X4R and mP2X4R expression in 1321N1 cells, [Ca^2+^]_i_ was measured in single cells using a Fura2-AM ratio imaging system and an inverted fluorescence microscope (ECLIPSE TE2000-U: Nikon, Tokyo, Japan) equipped with a Xenon-lamp (Xe75W; Nikon) and band-pass filters of 340 nm and 380 nm^49^. The emission fluorescence was measured at 510 nm. Image data were detected using Aquacosmos (Hamamatsu Photonics, Hamamatsu, Japan), and [Ca^2+^]_i_ was expressed as the ratio of fluorescence intensities at 340 nm and 380 nm. ATP (10 μM) was applied for 30 sec to rP2X4R- or mP2X4R-expressing 1321N1 cells, and the cells were pretreated with NP-1815-PX for 10 min.

### Cultured microglial cells

Primary cultured microglia were prepared according to a previously described method^50^,^51^. Microglial cells were used for the Ca^2+^ imaging assay in the Fura2-AM ratio imaging system described above. Following addition of 50 μM ATP for 30 sec, the microglial cells were pretreated with NP-1815-PX (0.3 and 1 μM) for 10 min. The cells were pre-incubated with ivermectin (3 μM) for 3 min prior to ATP application. In BV-2 cells (a cell line of mouse microglia), NP-1815-PX (1 μM) was pretreated for 10 min before application of BzATP (300 μM) for 30 sec.

### Animals

Male C57BL/6 (10 weeks old; Clea Japan, Tokyo, Japan) were used for the hot-plate and tail-flick tests, as well as traumatic nerve injury-induced allodynia. Female C57BL/6 mice (6 weeks old at start of experiment; Sankyo Labo Service Corporation, Toyama, Japan) were used to establish the herpetic pain model. Neonatal Wistar rats (Kyudo, Saga, Japan) were used for experiments in primary cultured microglia. The animals were housed under controlled temperature (22 ± 1 °C) and humidity (55 ± 10%). The room was lighted from 7:00–8:00 AM to 7:00–8:00 PM. Food and water were freely available. HSV-1 inoculation and behavioral experiments were performed in the infection room of the Molecular Genetics Research Center, Toyama Medical and Pharmaceutical University (Toyama, Japan). Other behavioral tests were performed at Kyushu University. The animal experiments were conducted in accordance with the Guiding Principles for the Care and Use of Laboratory Animals approved by the Committee for Animal Experiments at Toyama Medical and Pharmaceutical University and Kyushu University.

### Intrathecal injection of drugs

Under 2% isoflurane anesthesia, a 30-gauge needle attached to a 25-μl Hamilton syringe was inserted into the intervertebral space between the fifth and the sixth spinal vertebrae in mice, as previously described^52^.

### Hot-plate and tail-flick tests

Mice were placed on a metal surface maintained at 49 and 52 °C. The latency to either lick or shake the hindpaw or jump was measured. Noxious heat-evoked tail flick responses were detected by the application of radiant heat (50 V) (Ugo Basile, Italy)^53^. These behavioral assays were done 120 min after intrathecal administration of NP-1815-PX.

### Rotarod test

Each mouse was trained to run in a rotarod until it could remain there for 60 s without falling^53^. The mice were then evaluated in a rotarod test for 60 s 120 min after intrathecal injection of NP-1815-PX (30 pmol/5 μl).

### Traumatic nerve injury model

Under isoflurane (2%) anesthesia, the unilateral L4 spinal nerve was carefully isolated and cut as described previously^13^. To assess mechanical allodynia, calibrated von Frey filaments (0.02–2.0 g, North Coast Medical) were applied to the plantar surfaces of the hindpaw of mice, and the 50% paw withdrawal threshold was determined^13^. NP-1815-PX (30 pmol/5 μl) or PBS (5 μl) was administered intrathecally immediately after the behavioral measurement on day 7 after nerve injury, and the paw withdrawal threshold was measured for 360 min.

### Herpetic pain model

Mice were inoculated with HSV-1 as previously described^17^,^30^. Briefly, the epidermis of the right shin was scarified with a 27-gauge needle, and a 10-μl HSV-1 suspension (7401H strain, 1 × 10^6^ plaque-forming units) was topically applied to the scarified skin. Skin lesions were scored as follows: 0 = no lesions; 2 = one or two vesicles on the back; 4 = many vesicles on the back, the surrounding inoculated area, or both; 6 = mild herpes zoster-like lesions; 8 = apparent zoster-like lesions, paw inflammation, or both; and 10 = severe zoster-like lesions^17^,^30^. For the behavioral test for herpetic pain, dynamic allodynia was assessed as previously described^30^ by light stroking of the plantar surface of the hindpaw from the toe to the heel using an art paintbrush. Responses to stroking stimulation of the hindpaw were ranked as follows: 0, no response or moving the stimulated paw aside; 1, lifting of the stimulated paw toward the abdomen; 2, flinching or licking of the stimulated hindpaw. Stimulation was applied six times at intervals of several seconds, and the average value served as the pain-related score. Mice with a ≥ 0.5 pain-related score were considered to have allodynia. NP-1815-PX (10 or 30 pmol/5 μl) and TrkB-Fc (100 ng/5 μl) chimeric protein (R&D Systems) was intrathecally administered twice and once, respectively, daily from day 5 to 7 after HSV-1 inoculation.

### Immunohistochemistry

Immunohistochemistry was performed as previously described^13^. Transverse spinal cord (L4-5 segments) sections (30 μm) were incubated with the primary antibodies against P2X4R (rabbit polyclonal anti-P2X4R, 1:2000) kindly provided by Prof. Francois Rassendren (Université Montpellier, France), or cell markers; microglia, CD11b (rat monoclonal anti-CD11b, 1:1000, Serotec, Oxford, UK) and ionized calcium-binding adapter molecule-1 (Iba1) (rabbit polyclonal anti-Iba1, 1:2000, Wako, Osaka, Japan); astrocytes, glial fibrillary acidic protein (GFAP) (rat monoclonal anti-GFAP, 1:1000, Millipore, Darmstadt, Germany); neurons, neuronal nuclei (NeuN) (mouse monoclonal anti-NeuN, 1:200, Millipore, Darmstadt, Germany) and microtubule-associated protein-2 (MAP2) (mouse monoclonal anti-MAP2, 1:1000, Millipore, Darmstadt, Germany). The sections were incubated with the fluorescent conjugated secondary antibodies (Alexa Fluor 488 or 546, 1:1000, Invitrogen, Carlsbad, CA, USA). The sections were mounted with Vectashield (Vector Laboratories, Burlingame, CA, USA). Fluorescent images were obtained with a confocal microscope (LSM 5 Pascal; Carl Zeiss, Jena, Germany) and analyzed with Zeiss LSM Image Brower (Carl Zeiss, Jena, Germany).

### Real-time quantitative polymerase chain reaction

Total RNA from the third to sixth lumbar spinal cord was extracted using Trisure (Bioline, London, UK), according to the manufacturer’s protocol. The amount of total RNA was quantified using a Nanodrop spectrophotometer (Nanodrop, Wilmington, DE, USA). For reverse transcription, 100 ng of total RNA was transferred to the reaction with Prime Script reverse transcriptase (Takara, Kyoto, Japan) and random 6-mer primers. Quantitative polymerase chain reaction was carried out with Premix Ex Taq (Takara, Kyoto, Japan) using a 7500 real-time polymerase chain reaction system (Applied Biosystems, Foster City, CA, USA) according to the manufacturer’s specifications, and the data were analysed by 7500 System SDS Software 1.3.1 (Applied Biosystems Foster City, CA, USA) using the standard curve method. All values were normalized with to GAPDH (glyceraldehyde-3-phosphate dehydrogenase) expression. TaqMan probe, forward primer and reverse primer used in this study were designed as follows.

Iba1: 5′-FAM-CAGGAAGAGAGGCTGGAGGGGATCAA-TAMRA-3′ (probe), 5′-GATTTGCAGGGAGGAAAAGCT-3′ (forward primer), 5′-AACCCCAAGTTTCTCCAGCAT-3′ (reverse primer). P2X4R: 5′-FAM- CAATGAGCAACGCACACTCACCAAGG-TAMRA-3′ (probe), 5′-ACAACGTGTCTCCTGGCTACAAT-3′ (forward primer), 5′-GTCAAACTTGCCAGCCTTTCC-3′ (reverse primer). BDNF: 5′-FAM-CACTTCCCGGGTGATGCTCAGCA-TAMRA-3′ (probe), 5′-GCCCAACGAAGAAAACCATAAG-3′ (forward primer), 5′-TGTTTGCGGCATCCAGGTA-3′ (reverse primer). KCC2: 5′-FAM-CCTCTGCCTGGCCCTCATGTTCAT-TAMRA-3′ (probe), 5′-ATTTCGCTATTACCACTGGACTCTCT-3′ (forward primer), 5′-CCCGGTACTCGATGTACTTATAAATG-3′ (reverse primer). GAPDH: 5′-FAM-ACCACCAACTGCTTAGCCCCCCTG-TAMRA-3′ (probe), 5′-TGCCCCCATGTTTGTGATG-3′ (forward primer), 5′-GGCATGGACTGTGGTCATGA-3′ (reverse primer).

### Statistical analysis

Statistical analyses were performed using Student’s t test (Fig. 3b,c), Mann-Whitney U test (Fig. 6a), Friedman test (Fig. 4a,b), Kruskal-Wallis test (Fig. 5a) or one-way ANOVA with Dunnett’s test (Fig. 4c), Bonferroni’s Multiple Comparison test (Figs 2b and 3a) or Dunn’s test (Figs 5c,d and 6c–f) using GraphPad Prism 5.04 software. Differences were considered significant at P < 0.05.

## Additional Information

**How to cite this article**: Matsumura, Y. *et al*. A novel P2X4 receptor-selective antagonist produces anti-allodynic effect in a mouse model of herpetic pain. *Sci. Rep.* **6**, 32461; doi: 10.1038/srep32461 (2016).
